# Expertise Modulates Students’ Perception of Pain From a Self-Perspective: Quasi-Experimental Study

**DOI:** 10.2196/10885

**Published:** 2019-01-23

**Authors:** Sareh Said Yekta-Michael, André Schüppen, Arnim Johannes Gaebler, Jens Ellrich, Jan Willem Koten

**Affiliations:** 1 Department of Conservative Dentistry, Periodontology and Preventive Dentistry RWTH Aachen University Aachen Germany; 2 Interdisciplinary Center for Clinical Research RWTH Aachen University Aachen Germany; 3 Department of Psychiatry, Psychotherapy and Psychosomatics Medical Faculty RWTH Aachen Aachen Germany; 4 Medical Faculty Friedrich-Alexander-University Erlangen-Nuremberg Erlangen-Nuremberg Germany; 5 Institute of Psychology University of Graz Graz Austria

**Keywords:** medical education, virtual reality, questionnaires, physician

## Abstract

**Background:**

Perception of stimuli presented in a virtual dentistry environment affects regions of the brain that are related to pain perception.

**Objective:**

We investigated whether neural correlates of virtual pain perception are affected by education in dentistry.

**Methods:**

In this functional magnetic resonance imaging study, a sample of 20 dental students and 20 age-matched controls viewed and listened to video clips presenting a dental treatment from the first‐person perspective. An anxiety questionnaire was used to assess the level of dental anxiety. Neural correlates of pain perception were investigated through classic general linear model analysis and in-house classification methods.

**Results:**

Dental students and naïve controls exhibited similar anxiety levels for invasive stimuli. Invasive dentistry scenes evoked a less affective component of pain in dental students compared with naïve controls (*P*<.001). Reduced affective pain perception went along with suppressed brain activity in pain matrix regions including the insula, anterior cingulate cortex, and basal ganglia. Furthermore, a substantial reduction of brain activity was observed in motor-related regions, particularly the supplementary motor area, premotor cortex, and basal ganglia. Within this context, a classifier analysis based on neural activity in the nucleus lentiformis could identify dental students and controls on the individual subject level in 85% of the cases (34 out of 40 participants, sensitivity=90%, specificity=80%).

**Conclusions:**

Virtual dental treatment activates pain-related brain regions in controls. By contrast, dental students suppress affective and motor-related aspects of pain. We speculate that dental students learn to control motoric aspects of pain perception during their education because it is a prerequisite for the professional manual treatment of patients. We discuss that a specific set of learning mechanisms might affect perceived self-efficacy of dental students, which in turn might reduce their affective component of pain perception.

## Introduction

### Background

The quality of dentistry relies on the extent to which dentists can accurately plan, perform, and perceive motor actions. Planning performance and perception of potentially hurtful motor actions affect brain regions that are related to working memory and pain perception [1,2].

Pain is a variegated feeling, and it has a multidimensional nature with sensory-discriminative, aﬀective-motivational, motoric, and cognitive components [2-7]. Currently, neural correlates of orofacial pain are investigated through functional magnetic resonance imaging (fMRI) [8,9]. The affective component of pain is a subjective feeling that is often measured with standardized psychological scales that report the unpleasantness of stimuli. It is not only perceived when subjects are treated with physically painful stimuli but also when individuals are confronted with psychologically painful images or videos. However, the ability to perceive the affective component of pain is modulated by the professional training of a subject. Viewing hurting scenes induces affective component of pain in controls, whereas this is less the case for medical doctors [10,11]. fMRI studies show that medical doctors suppress the pain matrix when they view painful actions executed on others, whereas this is not the case for controls [10]. The pain matrix includes the thalamus, SI and SII, insula, as well as anterior cingulate cortex (ACC) [12-14]. In addition, cortical and subcortical motor regions including the basal ganglia are directly responsive to noxious stimuli [13,15]. Some of these cortical and subcortical motor regions show a nociceptive somatotopic organization [16].

Further, electroencephalogram studies suggest that early and late signal components show dissimilar behavior when controls view painful scenes, whereas this is not the case for doctors [11].

In addition, imaging studies showed that doctors and controls exhibit similar empathy, emotional contagion, and interpersonal reactivity scores [10,11].

Statistically valid differences between the 2 groups are exclusively found for scales that measure affective and sensory aspects of pain. From these neuroimaging studies, we have to conclude that affective and sensory aspects of pain for others may be modulated by education, whereas empathy for others as measured with several accepted scales is not. This leads to the somewhat counterintuitive conclusion that doctors maintain empathy for others, whereas suppressing neural correlates of pain for others [10,11,17]. We do not think that more research in the field of empathy is needed simply because previous empathy measurers failed to show differences between doctors and controls.

However, observed differences in affective pain perception for others might be linked to how doctors perceive pain from a self-perspective. Furthermore, one might speculate that differences in pain perception originate from medical training and occur in medical school.

### Objectives

In this paper, we investigate the differences of brain activations during virtual dental treatment in dentistry students and controls. We choose to investigate this through classic general linear model (GLM) analysis and in-house classification methods. Classification methods may have some advantages over classic analysis. First, one can specifically test whether hypothetical brain regions identified in other studies may in fact contribute to the identification of different neuropsychological states. Within this context, we focused on previously reported coordinates that were related to different components of pain perception [13]. Second, although classic fMRI methods only focus on univariate measures of brain activity, classification methods can identify the multivariate interplay among brain regions. These multivariate aspects of brain activity may be better predictors of a certain neuropsychological state as univariate measures. Finally, classification methods may isolate brain regions that are essential for a specific function.

We hypothesize that watching dentistry scenes from a self-perspective may induce brain activity in (affective and motoric aspects) pain-related regions in controls, whereas this is less the case for dental students. We expect that dental students learn to control the motoric aspects of pain during their education because it is a prerequisite for manual treatment [1].

## Methods

A total of 40 healthy (20 dental students, 20 controls), right-handed male volunteers (average age 28 years, SD 9) participated in this fMRI study [18] (Multimedia Appendix 1).

All subjects had been dentally treated in the recent past and had participated in former fMRI experiments. Controls were selected in the course of dental routine checkups in the Department of Conservative Dentistry (RWTH Aachen University). Dental students were selected 1 year before graduating. All volunteers gave their approval to the experimental conditions in written form.

Procedures were approved by the local ethics committee of University Hospital of Aachen, and all volunteers agreed to the World Medical Association Declaration of Helsinki (1964) and subsequent amendments.

**Figure 1:**
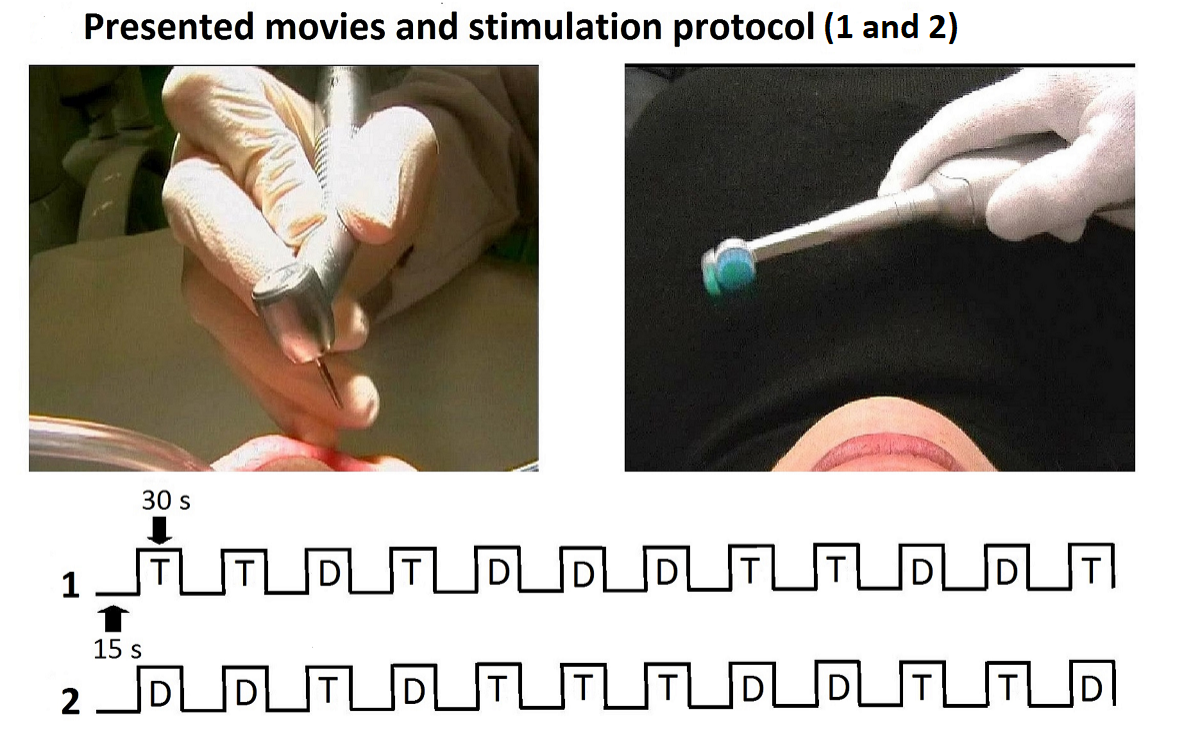
Stimulation protocol. Drilling movies presented a medical glove–wearing hand with a dental handpiece drilling a tooth in the right lower jaw. Toothbrush movies displayed the same gloved-hand using an electric toothbrush. Every single movie was presented 12 times in counterbalanced order for 30 seconds and separated by 12 resting baseline conditions that lasted 15 seconds. Both movies were presented in a randomized fashion to the volunteers.

The participants viewed and listened to drilling and toothbrush movies (Figure 1). The subjects were instructed to imagine the dental treatment from the perspective of a patient (first-person perspective). The sound of drilling and toothbrush movies was the same. The total duration of this procedure was 9 min.

### Anxiety Questionnaire

The Hierarchical Anxiety Questionnaire was used to assess the intensity of dental anxiety [19]. On the basis of an overall score ranging from 11 to 55, participants can be categorized into low anxious (<30), moderately anxious (31-38), and highly anxious (>38) groups.

### Pain Perception Questionnaire

The Pain Perception Scale is a common standard instrument for the study of pain, allowing standardized and multifaceted quantification of pain experience. By default, it contains 19 sensory and 14 affective descriptions of pain. All the subjects were assigned to rank each description for drilling and tooth brushing on a 4-point scale immediately after the fMRI session [20].

### Functional Magnetic Resonance Imaging Analysis

Scanning was performed by a Philips 3-Tesla magnetic resonance imaging *Model Achieva* (Philips Medical Systems). Axial slices were oriented toward the anterior-posterior commissure. A T2*-weighted echo planar imaging sequence was obtained for functional images: echo time 30 ms, repetition time 2800 ms, 32 interleaved slices (3.5 mm thick), flip angle of 90 degrees, field of view of 220 mm, voxel size of 3.75×3.75×3.5 mm, and 64×64 matrix. Functional data were imported into the Statistical Parametric Mapping (SPM) toolbox and coregistered with the high-resolution anatomical scan of the subject that was obtained in the same session. Preprocessing steps included the following: realignment, normalization to Montreal Neurological Institute (MNI) space, spatial smoothing (8 mm), and high-pass filtering (128 seconds). First-level beta weights were obtained by modeling the canonical hemodynamic response function within a GLM approach. Beta weights were used to contrast experimental conditions and experimental groups on a second level using a 2-sample *t* test. Furthermore, 2 contrasts were reported. First, we wanted to know if controls exhibit higher brain activity compared with dental students when the toothbrush condition was subtracted from the drilling condition (drill_C_−toothbrush_C_)−(drill_DS_−toothbrush_DS_). The latter contrast was liberally masked with the contrast drill_C_−toothbrush_C_ to avoid spurious activations. Next, we wanted to know if dental students exhibit higher brain activity compared with controls when toothbrush was subtracted from drilling. The latter contrast was masked with the drill_DS_−toothbrush_DS_. We thresholded contrasts at *P*<.001. Subsequently, we performed a Monte Carlo–based cluster threshold estimation procedure to correct for multiple testing. Next, beta contrast weights (drill−toothbrush) were extracted from 40 pain relevant cortical systems reported in a meta-analysis [13]. Extracted beta weights were subjected to a support vector machine analysis.

### Classifier Analysis

As information-based procedure to determine differences in brain activity between both groups on the individual subject level, we conducted a classifier analysis. Therefore, a modified support vector machine algorithm with a leave-one-out cross-validation was applied [21].

#### Region of Interest Definition and Feature Generation

Beta contrast weights (drilling−toothbrush) were extracted from pain-relevant brain regions reported in a meta-analysis [13]. For this purpose, structural scans were segmented into gray and white matter using the standard tools as available in the SPM software package. Regions of interest were defined, centering 4-mm diameter spheres on MNI coordinates of 40 pain-relevant brain regions reported in the abovementioned meta-analysis. Beta weights were extracted only from those voxels within the sphere which were found within the gray matter. This method avoids the extraction of spurious beta weights. Next, we averaged the beta weights per region. We subtracted beta weights of the toothbrush conditions from drill conditions for every region and subject. Functional masking of the beta weights was not needed because beta contrast weights of the drilling were positive and larger than the respective beta weights of the toothbrush conditions in all regions in all subjects.

#### Feature Selection

To consider only the most discriminating features for the classifier analysis, an information-based feature selection was applied. The discriminative power of a feature was defined as the absolute value of the Kendall tau correlation coefficient [22], which measured the correlation between a feature and the group indicator (−1 for controls, +1 for dental students). Thus, a positive correlation coefficient indicates that the feature (ie, the regional brain activity) increases in dental students compared with controls, whereas a negative correlation coefficient indicates that the feature decreases in the dental students compared with controls.

In each fold of the leave-one-out cross-validation, the features obtained from the n-1 remaining subjects were ranked according to their absolute value of the Kendall tau rank correlation coefficient and the feature, which exhibited highest relation to the group indicator, was selected.

#### Classifiers

The selected features were subjected to the classifier analysis applying support vector machines with a linear kernel [23]. Therefore, the support vector machine yielded a maximal-margin hyperplane in the feature space, which separated the groups in the respective training dataset. Classification was tested in the left-out sample. The limited number of subjects was lent to the leave-one-out cross-validation method to investigate the generalizability of the classification results. Importantly, this cross-validation encompassed the feature selection as well as the classifier. Accuracy (percentage of all participants detected correctly), sensitivity (percentage of dental students detected correctly), and specificity (percentage of controls detected correctly) quantified classification performance.

## Results

After the fMRI session, all controls and dental students declared that they could imagine being treated by a dental drill from a first-person perspective.

### Anxiety Questionnaire

From the 40 individuals under study, 12 controls and 10 dental students were categorized as low anxious, 8 controls and 8 dental students were categorized as moderately anxious, and 2 dental students were categorized as highly anxious (average controls 29, average dental students 30).

### Pain Perception Questionnaire

**Figure 2:**
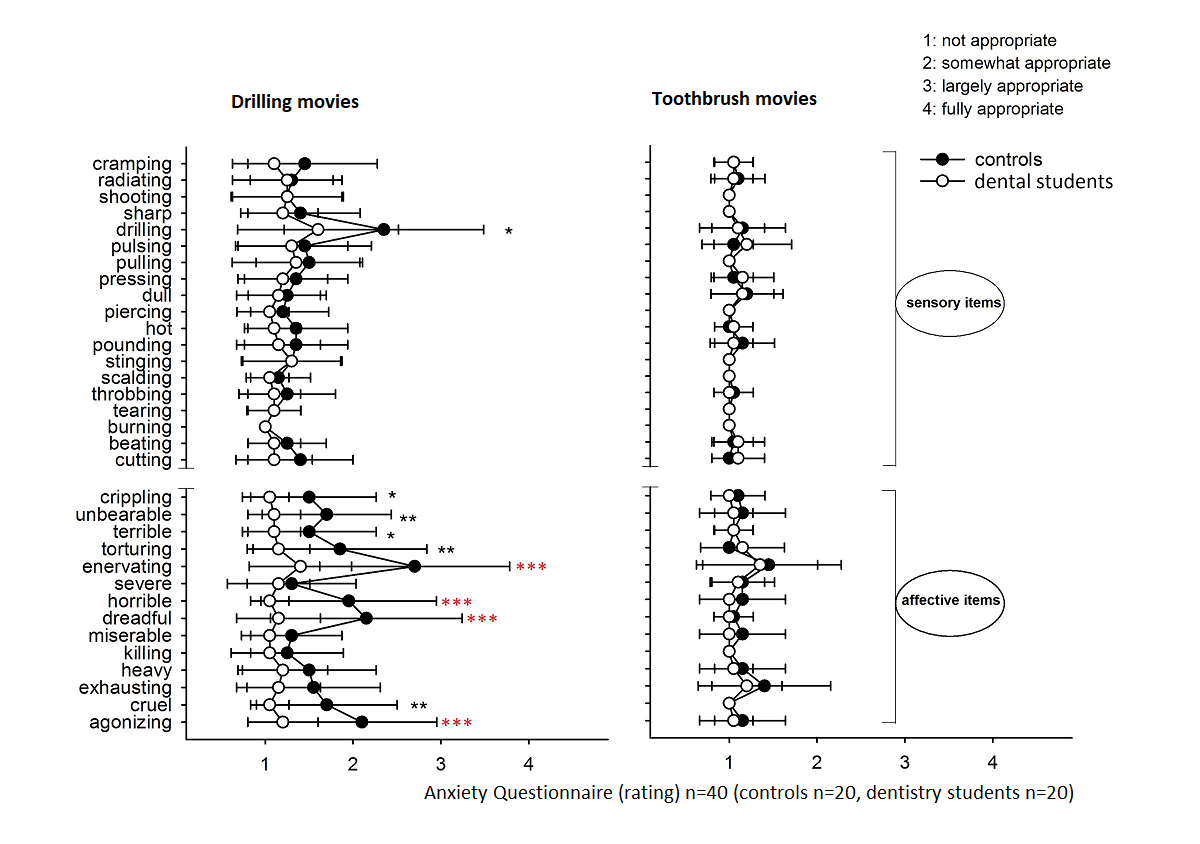
Anxiety Questionnaire. Comparison controls versus dental students (mean ± standard error of the mean, each group n=20; 1: not appropriate, 2: somewhat appropriate, 3: largely appropriate, 4: fully appropriate). Asterisks indicate significant differences between the ratings for dental students and controls for the drilling movies (one asterisk denotes *P*<.05, double asterisks denote *P*<.01, triple asterisks denote *P*<.001, *t* test). Red Asterisks: Bonferroni threshold.

We chose to analyze data with a very conservative approach. For every condition, 33 *t* tests were executed according to the number of items in the questionnaire. Next, the critical Bonferroni threshold .05/33 was estimated. The summary statistic based on 2 sample *t* tests is visualized (Figure 2). No significant differences between dental students and controls were observed for toothbrush, but large differences were observed for drilling. For drilling, significant differences between dental students and controls were observed for affective pain scales but not for sensory pain scales.

Dental students showed significantly lower pain than controls for 4 affective items, namely agonizing, dreadful, horrible, and enervating (*P*<.001, Bonferroni).

**Figure 3:**
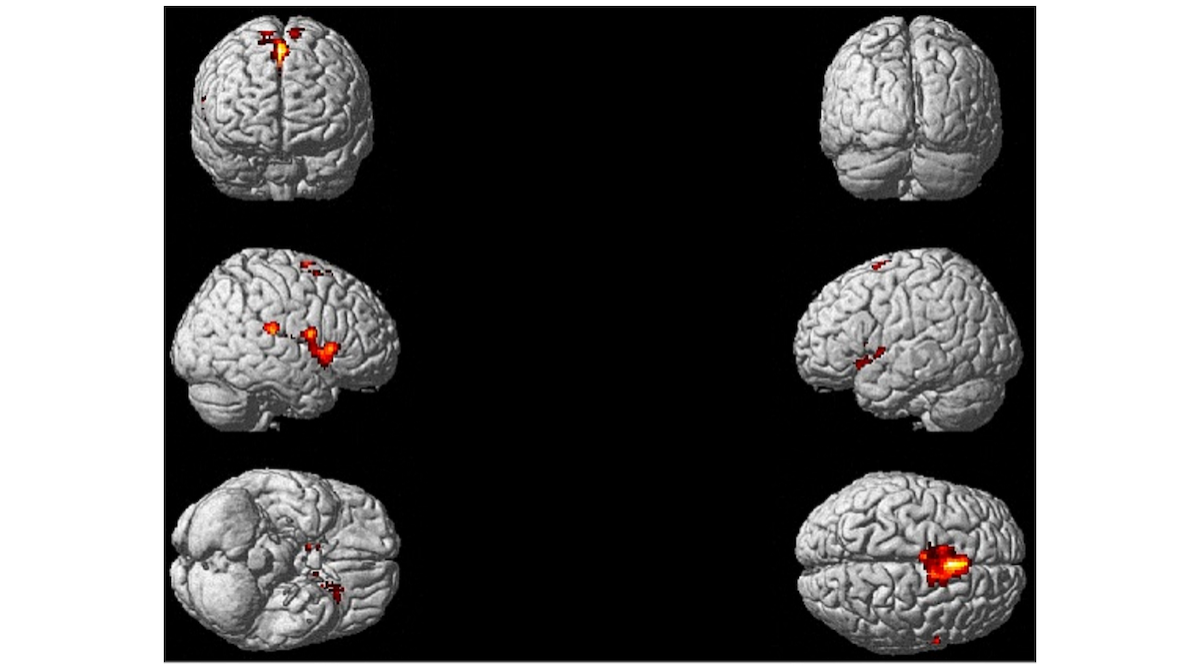
Brain activity. Controls exhibit higher brain activity compared with dental students when the toothbrush conditions were subtracted from the drilling conditions (drillC-toothbrushC)-(drillDS-toothbrushDS). The latter contrast was liberally masked with (drillNC-toothbrushC). Contrasts were thresholded at P<.001 with k=156.

**Figure 4:**
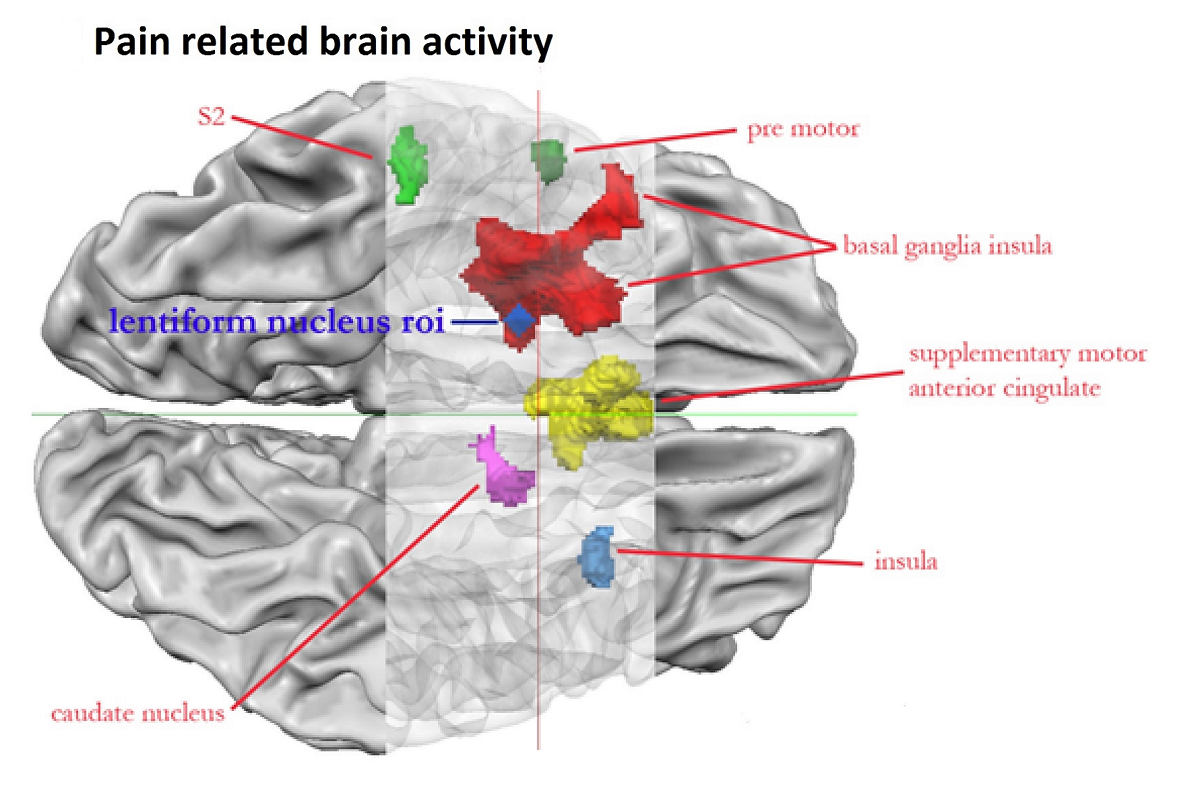
Region of interest (roi). Controls exhibit higher brain activity compared with dental students when the toothbrush conditions were subtracted from the drilling conditions (drillC-toothbrushC)-(drillDS-toothbrushDS). The latter contrast was liberally masked with (drillC-toothbrushC). Contrasts were thresholded at *P*<.0005 with k=125.In addition, we visualize the roi that leads to best classification results.

Neural aspects of pain perception were not higher in controls compared with dental students when toothbrush was shown. Moreover, neural aspects of pain perception were not higher in dental students compared with controls when drilling was compared with toothbrush (empty contrast). By contrast, neural aspects of pain perception were higher in controls than in dental students when drilling was compared with toothbrush (Figures 3 and 4; Multimedia Appendix 2).

### Functional Magnetic Resonance Imaging Analysis

Results (*P*<.001 using a conservative cluster threshold of *k*=159; Figure 3) revealed that brain activations were organized around sensory-motor and limbic-affective systems. The sensory motor system included larger parts of the supplementary motor area as well as SII. Furthermore, limbic-affective structures included the thalamus basal ganglia as well as larger parts of the posterior and frontal insula and ACC. It is clear that some structures can be part of more systems. For instance, the basal ganglia are part of the motor loop. We used the standard SPM preprocessing pipeline that employs rather crude smoothing kernels (8 mm) and rough brain alignment methods. This may lead to spurious activations even when conservative cluster thresholds are used. In a next step, we decided to threshold our image at .0005 in combination with a less conservative cluster threshold (*k*=125). Some of the previously discussed activations disappeared. Unfortunately, the SPM brain rendering does not fully inform about subcortical and insular activations. For this reason, a ventral view of the activation is presented in Figure 4 using the brain voyager software.

In dental students, brain activity in a web of brain regions known as the pain matrix was suppressed. Remarkably enough, the difference between dental students and controls was not found in regions that are of core importance for the processing of the sensorial aspects of pain such as the SI. However, dental students showed a marked suppression of brain activity in regions related to the affective aspects of pain including the bilateral insula and bilateral ACC. In addition, motor-related aspects of pain perception were reduced in dental students. The latter included the premotor cortex as well as the nuclei of the basal ganglia and caudate nucleus (Figures 3 and 4). We want to stress that we could not identify brain regions that were activated in dental students but not in controls.

### Classifier Analysis

In the classifier analysis, we were able to classify 85% (34 participants of 40) of the participants on the basis of neural activity found in the most discriminative region in each fold of the cross-validation (sensitivity=90.0%, specificity=80.0%). It turned out that in each fold of the leave-one-out cross-validation, the neural activity in the right nucleus lentiformis (dark blue region in Figure 4) was selected as the most discriminative feature. Adding more regions to the classifier did not result in better classification performance.

## Discussion

### Principal Findings

In this study, we showed that dentistry students suppress pain-linked brain activity when brought into a virtual dental-treatment environment, whereas this is less the case for controls. In addition, we could identify dental students and controls on the basis of brain activity located in the lentiform nucleus.

In this study, affective items of Anxiety questionnaire clearly indicated a difference between controls and dental students. Controls perceived drilling as more unpleasant when compared with dental students. By contrast, no affective pain perception differences were observed for the toothbrush. In addition, both groups showed similar scores with regard to sensory aspects of pain regardless of the condition type under study. This indicates that dental students experience less affective component of pain than controls when viewing invasive dentistry scenes. The reduced pain perception of dental students correlated with the reduced brain activity of pain matrix regions. The latter included regions related to the somatosensory system as well as regions that have been related to affective aspects of pain perception including parts of the insula [24] and ACC [12,25]. We also observed reduced activity of dental students in subcortical and cortical motor regions including precentral gyrus (Broadman area 6) and the basal ganglia, particularly the lentiform nucleus. The observed differences in motor regions suggest that dental students suppress motoric components of pain when confronted with invasive stimuli. In short, most of our hypotheses were confirmed.

A difference between previous studies and this study is that we assessed affective pain perception from a self-perspective, whereas Cheng and Decety investigated affective pain from another perspective. Despite the obvious difference in perspective, we reproduce some important findings of Chen et al. In both studies, controls exhibited increased activity in ACC and the supplementary motor area when confronted with invasive stimuli [26]. However, Cheng observed that experts activate a frontal parietal system when confronted with invasive stimuli. They speculated that these increases in brain activation were linked to emotion regulation and theory of mind. We did not observe increased brain activity in dental students. It may be possible that observed differences between the 2 studies are because of the self-perspective versus other perspective. However, one should be careful with these kinds of speculations. In fact, the study of Cheng lacked a control condition from a self-perspective, whereas this study lacks a control condition from the other perspective.

This study also shows the benefits of classification approaches. We expected that a larger number of regions was needed to obtain sufficient classification rates [21,27]. However, in fact, only 1 region, namely the lentiform nucleus, was needed to classify controls and dental students on the basis of brain activity. This suggests that the latter region is of core importance in pain perception. Although conventional GLM analysis may be used to trace differences between groups, it is maybe not the ideal method to isolate brain regions that are of core importance for a specific function. Hence, classification methods may be a useful complement to conventional GLM methods.

Our findings suggest that affective and motoric components of pain suppression of dental students might possibly originate from dental school training. Recently, it has been suggested that distinct learning mechanisms affect pain expectancy, which in turn affects pain perception [28]. According to Peerdeman, pain perception can be affected by cognitive instructions, observational learning, and operant conditioning. The 3 learning mechanisms mentioned may modulate pain expectancy in medical students. Pain expectancy has been linked to activity in the insula [29] and basal ganglia [30]. Our results suggest that missing activity in the insula and basal ganglia in dental students reflects lower anticipation of pain. Furthermore, Peerdeman argues that the aforementioned learning mechanisms may affect self-efficacy expectancy. This is defined as the extent to which people can voluntary control aspects of pain. Neural correlates of self-efficacy have been linked to the nucleus lentiformis [31]. As demonstrated in this study, brain activity of this region predicts whether an individual belongs to the dental students or controls. As mentioned above, we speculate that maintaining motor control in the face of pain is an important ingredient of medical education. A previous study investigated the cognitive aspects of motor actions in a virtual dentistry environment in a sample of dentistry students [1]. However, the quality of the dentistry treatment may not only depend on the cognitive aspects of motor actions but also on emotional aspects of motor actions. The latter was the main object of our virtual-reality study, and we therefore think that it completes the previous motor study.

### Limitations

As a limitation, the sample size of this study is 40 subjects. Although this is perfectly normal for an fMRI study, one might argue that the power to detect effects is not very large. We would like to argue that we replicate important findings of our colleagues.

There are some limitations in our experimental design. First, it might be better to study the effect of hurtful scenes from both a self- and another perspective. Second, it might be better to study dental students at the end and beginning of their studies using a within-subject design. Finally, one might investigate if individual differences in pain perception of dental students are linked to their clinical performance [17]. In previous studies, subjects viewed hurtful actions inflicted on others, whereas in this study subjects viewed hurtful actions inflicted on the subject itself [8-11]. It is indeed possible that the expertise obtained during medical school leads to a suppression of pain from a self-perspective, which in turn might affect pain perception from another perspective. However, in principle, an opposite mechanism is possible, namely reduced pain perception for others leads to reduced pain perception for oneself. Unfortunately, it is not possible to identify the exact causal relation in this study.

### Conclusions

We conclude that dental students suppress affective and motor-related aspects of pain. The cognitive mechanisms that modulate pain expectancy and pain perception are poorly understood and deserve further investigation. A candidate mechanism is self-efficacy that may be linked to brain activity in the nucleus lentiformis.

## Appendix

Multimedia Appendix 1 Consolidated Standards of Reporting Trials (CONSORT)-flow diagram.

Multimedia Appendix 2 The statistics for brain coordinates.

## Abbreviations

ACC: anterior cingulate cortex
fMRI: functional magnetic resonance imaging
GLM: general linear model
MNI: Montreal Neurological Institute
SPM: Statistical Parametric Mapping
