# Supplementary figures and images for: Expertise Modulates Students’ Perception of Pain From a Self-Perspective: Quasi-Experimental Study
Source: J Med Internet Res. 2019 Jan 23;21(1):e10885. doi: 10.2196/10885 (PMC6364199; doi:10.2196/10885)

## CONSORT 2010 Flow Diagram

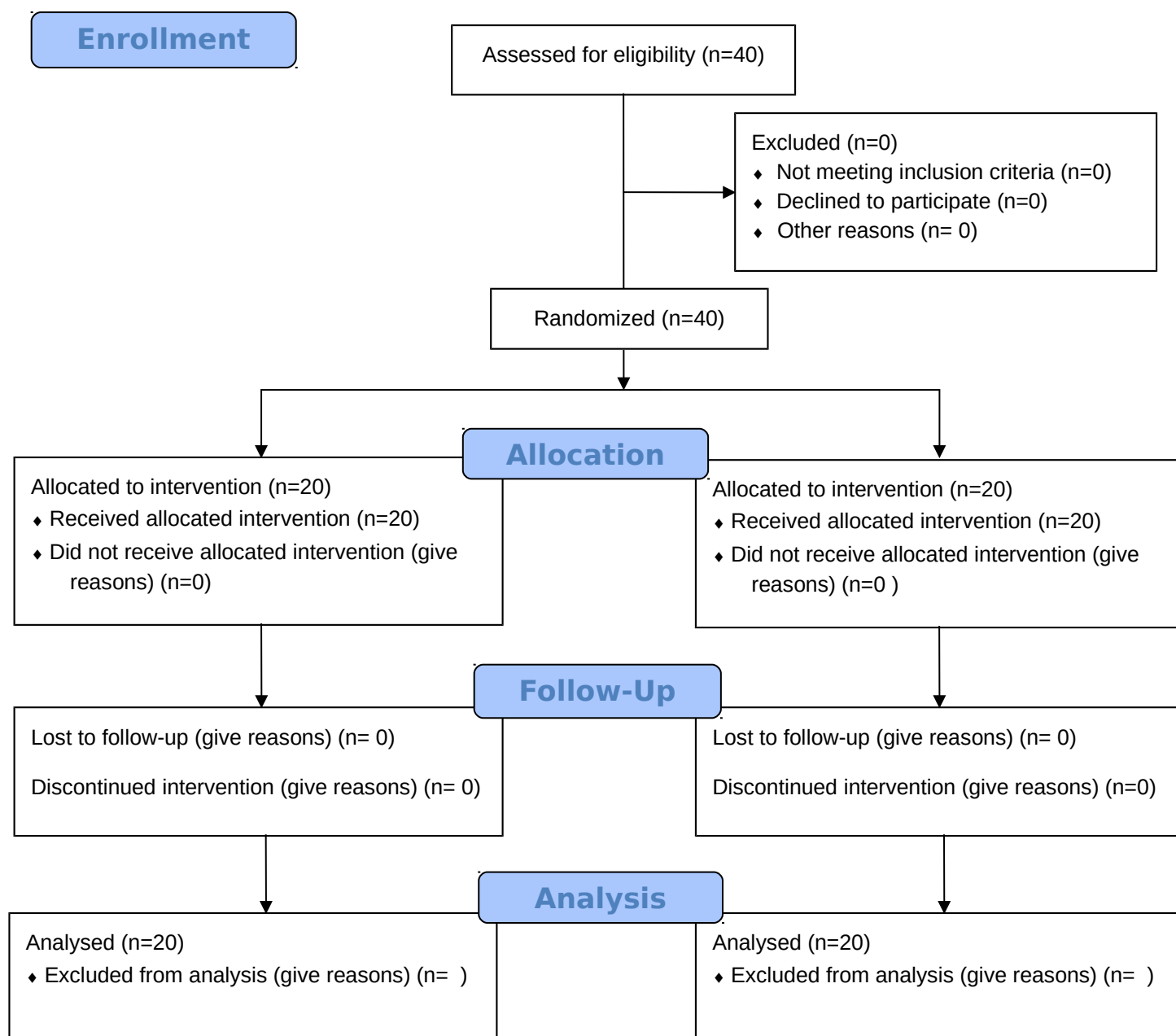

Supplement: Multimedia Appendix 1 [file jmir_v21i1e10885_app1.pdf]
