# Supplementary material for: Expertise Modulates Students’ Perception of Pain From a Self-Perspective: Quasi-Experimental Study
Source: J Med Internet Res. 2019 Jan 23;21(1):e10885. doi: 10.2196/10885 (PMC6364199; doi:10.2196/10885)
Supplement: Multimedia Appendix 2 [file jmir_v21i1e10885_app2.pdf]

Multimedia Appendix 2

1. This table reports the relevant statistic for a given brain coordinate as reported by SPM12 for the contrast (drill$_C$-toothbrush$_C$)-(drillDS-toothbrush$_{DS}$). The latter contrast was liberally masked with (drill$_C$-toothbrush$_C$). C=controls, DS=Dentistry students.

cluster				peak					
p(FEW-corr)	p(FDR-corr)	equivk	p(unc)	p(FWE-corr)	p(FDR-corr)	T	equivZ	p(unc)	x,y,z {mm}
0.000	0.000	2520,00	0.000	0.010	0.193	5.82	4.89	0.000	30 4 8
				0.027	0.205	5.46	4.66	0.000	24 -2 10
				0.032	0.205	5.39	4.61	0.000	34 -4 6
0.000	0.000	1495,00	0.000	0.068	0.275	5.08	4.41	0.000	2 12 56
				0.101	0.289	4.93	4.30	0.000	10 18 36
				0.151	0.350	4.76	4.19	0.000	0 24 52
0.000	0.000	1107,00	0.000	0.218	0.450	4.59	4.07	0.000	-16 -6 18
				0.353	0.465	4.35	3.90	0.000	-18 16 -4
				0.355	0.465	4.35	3.89	0.000	-30 22 -10
0.106	0.128	192,00	0.027	0.293	0.465	4.45	3.97	0.000	60 -28 18
				0.686	0.505	3.93	3.58	0.000	46 -32 18
0.065	0.096	234,00	0.016	0.514	0.465	4.14	3.74	0.000	8 28 10
				0.805	0.577	3.78	3.46	0.000	2 34 12
				0.836	0.604	3.73	3.42	0.000	4 24 18

Table shows 3 local maxima more than 8.0mm apart

Height threshold: T = 3.32, *P* = 0.001 (0.985)

Extent threshold: k = 159 voxels, *P* = 0.041 (0.158)
Expected voxels per cluster, <k> = 37.050
Expected number of clusters, <c> = 0.17
FWEp: 5.209, FDRp: Inf, FWEc: 1107, FDRc: 1107
FWHM = 15.5 15.1 12.9 mm mm mm; 7.8 7.5 6.5 {voxels}
